# Construction and Validation of a Necroptosis-Related Signature Associated With the Immune Microenvironment in Liver Hepatocellular Carcinoma

**DOI:** 10.3389/fgene.2022.859544

**Published:** 2022-04-11

**Authors:** Gongjun Wang, Baoning Ding, Libin Sun, Jing Guo, Shasha Wang, Wenqian Li, Yuqi Zhang, Jing Lv, Wensheng Qiu

**Affiliations:** ^1^ Department of Oncology, Affiliated Hospital of Qingdao University, Qingdao, China; ^2^ School of Statistics, Shandong University of Finance and Economics, Jinan, China

**Keywords:** liver hepatocellular carcinoma, necroptosis, prognosis, immune, tumor microenvironment

## Abstract

**Background:** Liver hepatocellular carcinoma (LIHC) is a widespread and often deadly neoplasm. There is increasing evidence that necroptosis mediates numerous tumor-associated behaviors, as well as the regulation of the tumor microenvironment, suggesting its use as a biomarker for tumor prognosis.

**Methods:** Data on mRNA expression and necroptosis regulators were acquired from the TCGA and KEGG databases, respectively. Clinical liver hepatocellular carcinoma (LIHC) patient data and information on the expression of necroptosis regulators were processed by unsupervised cluster analysis was performed on LIHC patients together with necroptotic regulator expression and, differentially expressed necroptosis-related genes (DENRGs) were identified by comparing the two clusters. A signature based on eight DENRGs was constructed and verified through independent data sets, and its relationship with the tumor microenvironment was investigated.

**Results:** Unsupervised cluster analysis demonstrated inherent immune differences among LIHC patients. In all, 1,516 DENRGs were obtained by comparison between the two clusters. In the training set, the final eight genes obtained by univariate, LASSO, and multivariate Cox regression were utilized for constructing the signature. The survival and receiver operating characteristic (ROC) curve achieved satisfactory results in both sets. The high-risk group was characterized by greater immune infiltration and poor prognosis. The results of survival analysis based on the expression of eight DENRGs further confirmed the signature.

**Conclusion:** We established and validated a risk signature based on eight DERNGs related to the tumor microenvironment. This provides a possible explanation for the different clinical effects of immunotherapy and provides a novel perspective for predicting tumor prognosis in LIHC.

## Introduction

Liver cancer is a common and deadly cancer (Sung et al., 2021). Liver hepatocellular carcinoma (LIHC) comprises 75–85% of primary liver cancers (Sung et al., 2021). The recommended curative therapy is surgical resection. However, the cancer is often advanced when diagnosed and resection may thus no longer be appropriate (Wei et al., 2019). Although there has been significant progress in LIHC treatment, due to its metastasis and recurrence, the median survival time of patients with advanced LIHC and the 5-years survival rate remains poor (Dutta and Mahato, 2017). There is, therefore, a need for the identification of biomarkers to facilitate the diagnosis and management of LIHC.

Necroptosis is a form of programmed cell death that occurs when apoptosis is blocked (Pasparakis and Vandenabeele, 2015). It has been implicated in involved in numerous processes, such as infection, liver disease, kidney injury, neurodegeneration, cardiovascular disease, and tumors (Dai et al., 2021). Necroptosis can modulate the immune response in tumors, and thus may have potential immunotherapeutic applications (Yatim et al., 2015; Aaes et al., 2016; Van Hoecke et al., 2018). On the one hand, necroptotic cancer cells release damage-associated molecular pattern (DAMP) molecules, cytokines, and chemokines into the tumor microenvironment, resulting in inflammatory, and tumor-modulating effects. On the other hand, necroptotic tumor cells also attract macrophages and dendritic cells, while stimulating effector T cells to infiltrate tumor tissues. Immunosuppression may be further enhanced by the activation of myeloid suppressor cells and tumor-associated macrophages (Sprooten et al., 2020). Necroptosis has also been reported to mediate sorafenib resistance (Liao et al., 2021) and tumor metastasis (Chen et al., 2021), and has been proposed as a prognostic marker in hepatocellular carcinoma (Yao et al., 2017). Thus, there is likely to be a close relationship between necroptosis and LIHC. However, there is as yet no systematic evaluation of a necroptosis-associated gene signature and how these genes may be related to the outcomes of LIHC patients.

Here, we investigated the construction and validation of a necroptosis-associated signature and its potential for prognosis prediction in LIHC patients, using various parameters including clinical features, and immune cell infiltration. These results contribute to an improved understanding of immunogenic cell death, cancer immunotherapy, and the application of prognostic markers.

## Materials and Methods

### Data Collection

Information on gene expression and the clinical data of LIHC patients were downloaded from The Cancer Genome Atlas (TCGA) database (https://portal.gdc.cancer.gov). If there was no available information on survival, the patient was not selected. One hundred and fifty-nine necroptotic regulators (hsa04217) were acquired from the Kyoto Encyclopedia of Genes and Genomes (KEGG) database (https://www.kegg.jp/). Ethical approval and informed consent were not applicable as the data are publicly available.

### Differentially Expressed Necroptosis-Related Genes

Unsupervised consensus clustering was applied to the LIHC patient data based on the expression of 159 necroptotic regulators, to identify specific distinct necroptosis-related patterns. The “ConsensuClusterPlus” package was used to assess the number and stability of the clusters, in accordance with the elbow method and Gap statistic (Wilkerson and Hayes, 2010). The R package “limma” was then used for the identification of the 1,516 DENRGs through cluster comparisons (Ritchie et al., 2015), using the criteria of false discovery rate (FDR) < 0.05 and fold change (FC) absolute value > 1.5.

### Screening of Candidate DENRGs and Determination of the Prognostic Signature

The 365 LIHC patients were allocated to either a training or validation set in a 1:1 ratio using R. The training set was used for subsequent analysis. DENRGs that were related to survival were examined by univariate Cox regression, and further selection was performed using the “least absolute shrinkage and selection operator” (LASSO) with the “glmnet” package to avoid overfitting (Engebretsen and Bohlin, 2019). Multivariate regression was then conducted to identify DENRGs suitable for use in the prognostic signature. The coefficients of the DERNGs used in the final signature were verified at the same time and were used to determine the risk scores of each of the patients in the training set, with the score determined as:

$$\text{Risk}\;\text{Score}\;=\sum_{i=0}^{n}\beta i\;\text{∗}\;Gi$$

βi is the coefficient of gene i in the multivariate analysis; Gi represents the expression of gene  i ; and n represents the total number of genes.

Using the median risk score, the patients were assigned to a high-risk or low-risk group. Kaplan–Meier (K-M) survival curves with the log-rank test were established to determine the difference in overall survival (OS) between the two groups. Receiver operating characteristic (ROC) curves were created for 1-year, 3-year, and 5-year OS using the “survival ROC” package, and the areas under the curves (AUCs) were determined. Principal component analysis (PCA) and T-distributed stochastic neighbor embedding (t-SNE) were applied for dimensionality reduction, using the “Rtsne” package in R.

### Validation of the Prognostic Signature

Data on the expression of genes used in the signature were then taken from the validation set and used for calculating risk scores, as described above. The individuals in the validation set were allocated to groups using the same method as the training set. The predictive capability of the signature was verified using ROC, K-M survival curves, PCA, and t-SNE.

### Development of Nomogram Integrating the DENRG-Based Signature and Clinical Variables

Multivariate Cox regression was conducted on the significant (*p* < 0.05) factors derived from the univariate analysis of the signature and clinical parameters in the training set. A prognostic nomogram was constructed with the “rms” package in R using the identified independent factors for OS prediction. The agreement between the actual OS and those predicted by the nomogram was evaluated by calibration curves.

### Functional Annotation of DENRGs

Gene Ontology (GO) annotation and KEGG pathway analyses were undertaken using the “clusterprofiler” package in R, with adjusted *p*-values <0.05 representing the significance threshold.

### Estimation of Immune Cell Infiltration

The “Single Sample Gene Set Enrichment Analysis” (ssGSEA) algorithm was used to measure the proportions of 16 immune cell types in the two groups. The infiltration of six cell types, namely, B cells, CD4^+^ T cells, CD8^+^ T cells, neutrophils, macrophages, and dendritic cells, were assessed in the TCGA samples and their association with the levels of genes used in the signature was determined using the TIMER algorithm (https://cistrome.shinyapps.io/timer/).

### Expression Analysis of DENRGs

Gene Expression Profiling Interactive Analysis (GEPIA) is a commonly used interactive website that compares gene expression differences between patients and normal controls (http://gepia.cancer-pku.cn/). The Human Protein Atlas (HPA) was used for qualitative comparison of IHC staining of liver cancer tissue with normal liver (http://www.proteinatlas.org/).

### Statistical Analyses

R (version 4.1.0) was used for all data analysis. Spearman’s and distance correlations were used to determine relationships between variables. Differences in survival curves were assessed by log-rank tests. Clinical parameters were compared using t-tests and chi-square tests, while Cox regression was used to measure survival-associated parameters. Two-tailed *p*-values <0.05 were considered statistically significant. The optimal cut-off values were established by the X-tile program (X-tile software version 3.6.1) (Camp et al., 2004) were used for the determination of gene expression levels.

## Results

### Overview of Necroptotic Regulators

**FIGURE 1:**
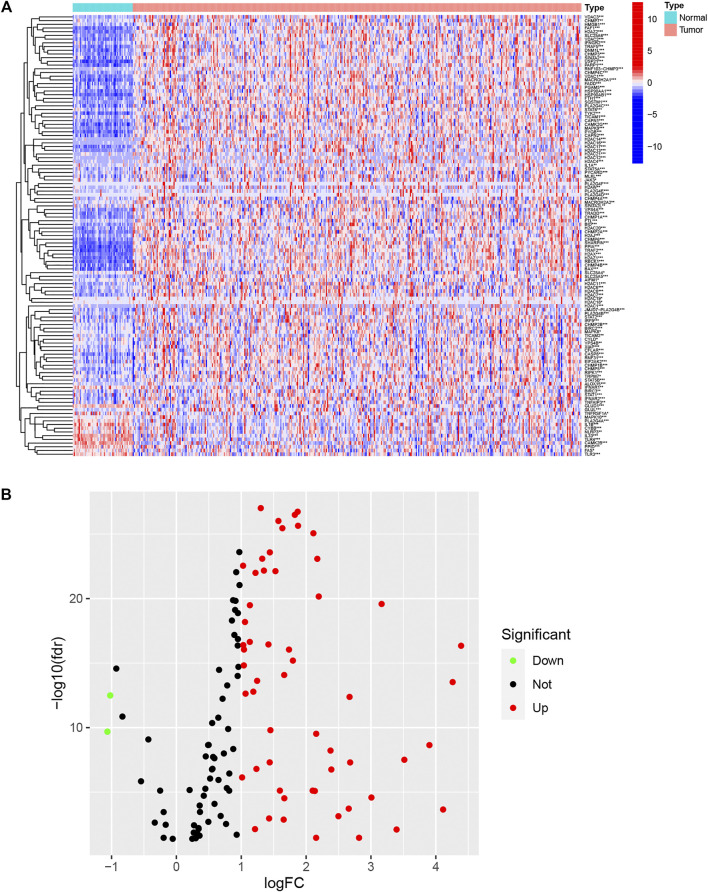
Expression of necroptotic regulators in LIHC and normal tissues. **(A)** Heatmap showing expression of necroptotic regulators between tumor and normal tissues. **(B)** Volcano plot showing fold changes of necroptosis regulators between tumor tissue and normal tissue. *p < 0.05, **p < 0.01, and ***p < 0.001.

A flow chart illustrating the investigation steps is given in Supplementary Figure S1. In total, the data of 374 LIHC cancerous and 50 non-cancerous tissues were obtained from the TCGA database and included in the study. One hundred and fifty-nine necroptotic regulators (Supplementary File S1) were identified from the KEGG database, and their expression between LIHC and normal tissue was found to differ significantly (Figure 1A). As seen in the volcano plot (Figure 1B), 56 of the regulators were up-regulated (log FC > 1), while only two were down-regulated in LIHC (log FC < −1). The results indicate that most necroptotic regulators may act as oncogenes in LIHC.

### DENRGs-Based Clusters and Determination of DENRGs

**FIGURE 2:**
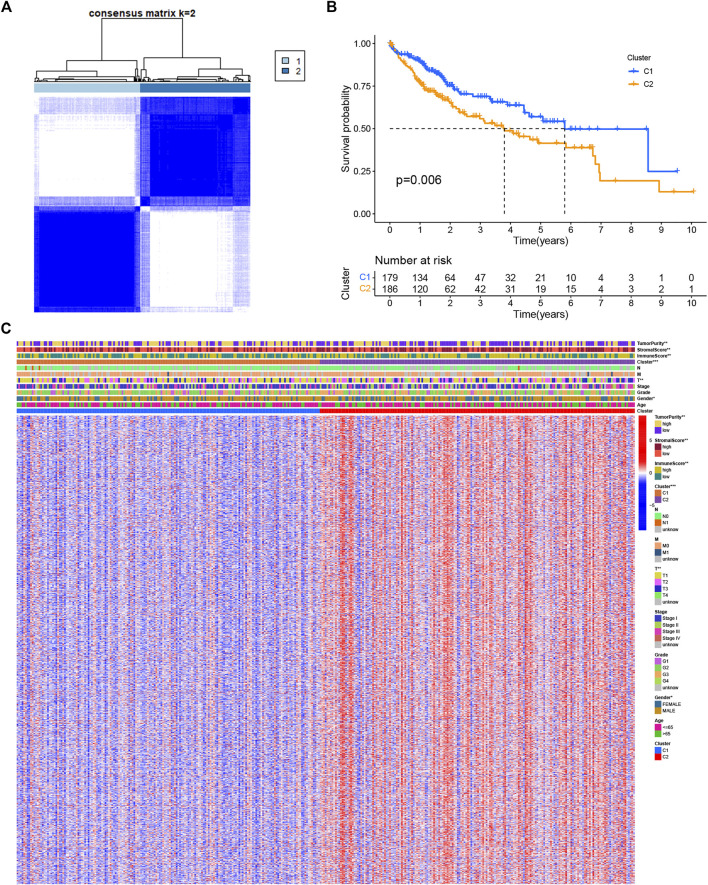
Clusters based on the expression of necroptotic regulators in LIHC. **(A)** Consensus matrix heatmap of LIHC patients when k = 2. **(B)** OS curves for the two clusters showing the association between necroptosis regulator-based clusters and OS. **(C)** Consensus clustering of clinical variables, immune status, and differentially expressed genes between the two necroptosis-related clusters. *p < 0.05, **p < 0.01, and ***p < 0.001.

To investigate the molecular heterogeneity of LIHC and identify patterns in LIHC patients based on necroptotic regulators, unsupervised consensus analysis was conducted on tumor samples, and different subgroups were established. The optimal cluster numbers (k) were calculated by setting the value of k from 2 to 9 (Supplementary Figure S2); it was found that k = 2 was the optimum value in terms of both within-cluster homogeneity and between-cluster heterogeneity (Figure 2A). Cluster 1 (C1) was associated with better OS, shown by K-M analysis (Figure 2B). A comparison between C1 and Cluster 2 (C2) yielded 1,516 DENRGs (fold change = 1.5) (Supplementary File S2). The heatmap of the distributions of the different clinicopathological features and immune scores between the clusters is shown in the panel below the heatmap (Figure 2C). Clinical variables, such as T stage, sex, and immune status differed between the clusters.

### Establishment of a Prognostic DENRGs Signature

**FIGURE 3:**
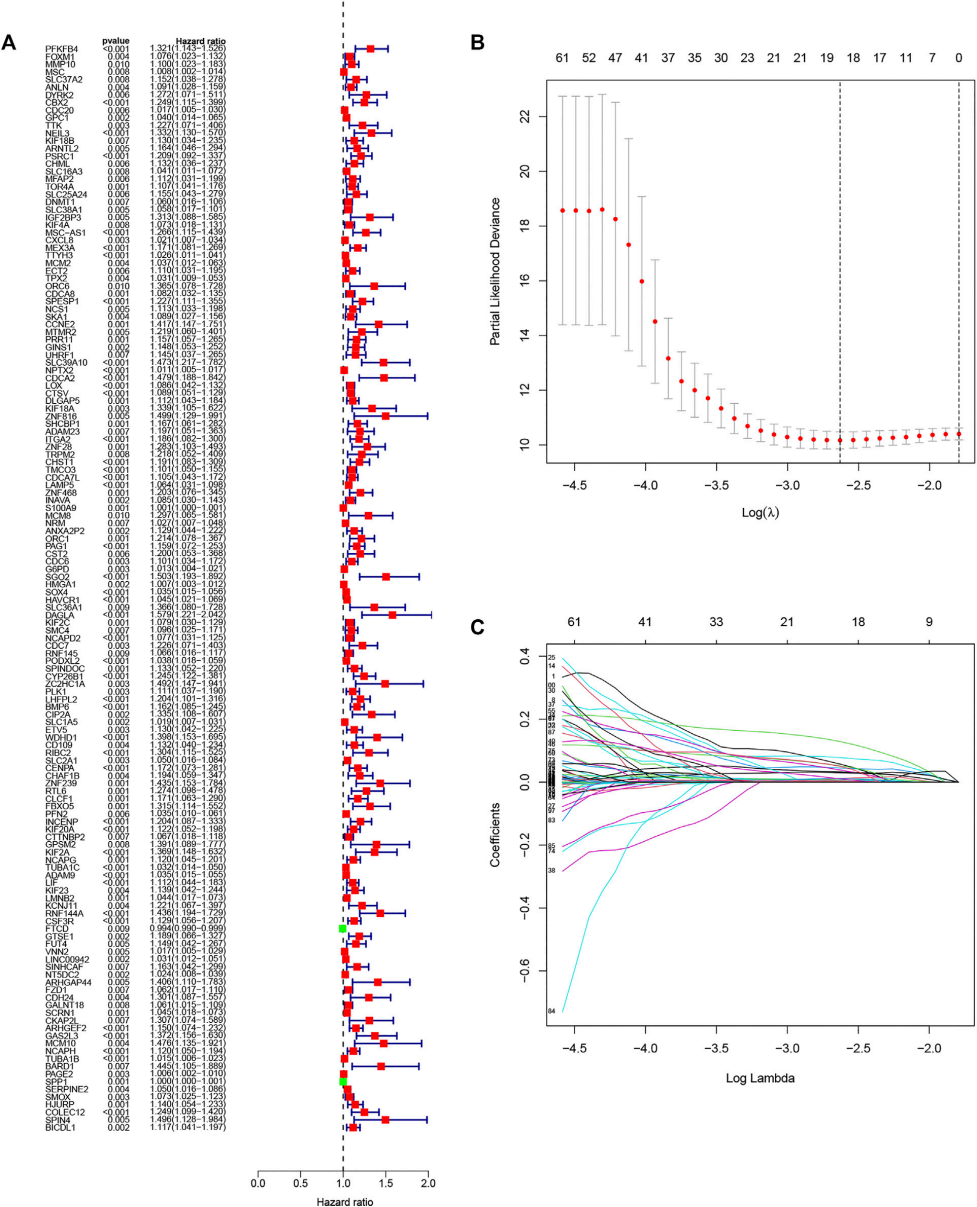
Selection of genes in the training set for inclusion in the signature. **(A)** Forest plot of univariate results showing 143 OS-related DENRGs (*p* < 0.05). **(B)** LASSO was used to screen OS-related DENRGs. **(C)** LASSO coefﬁcient proﬁles determined by the optimal lambda.

**FIGURE 4:**
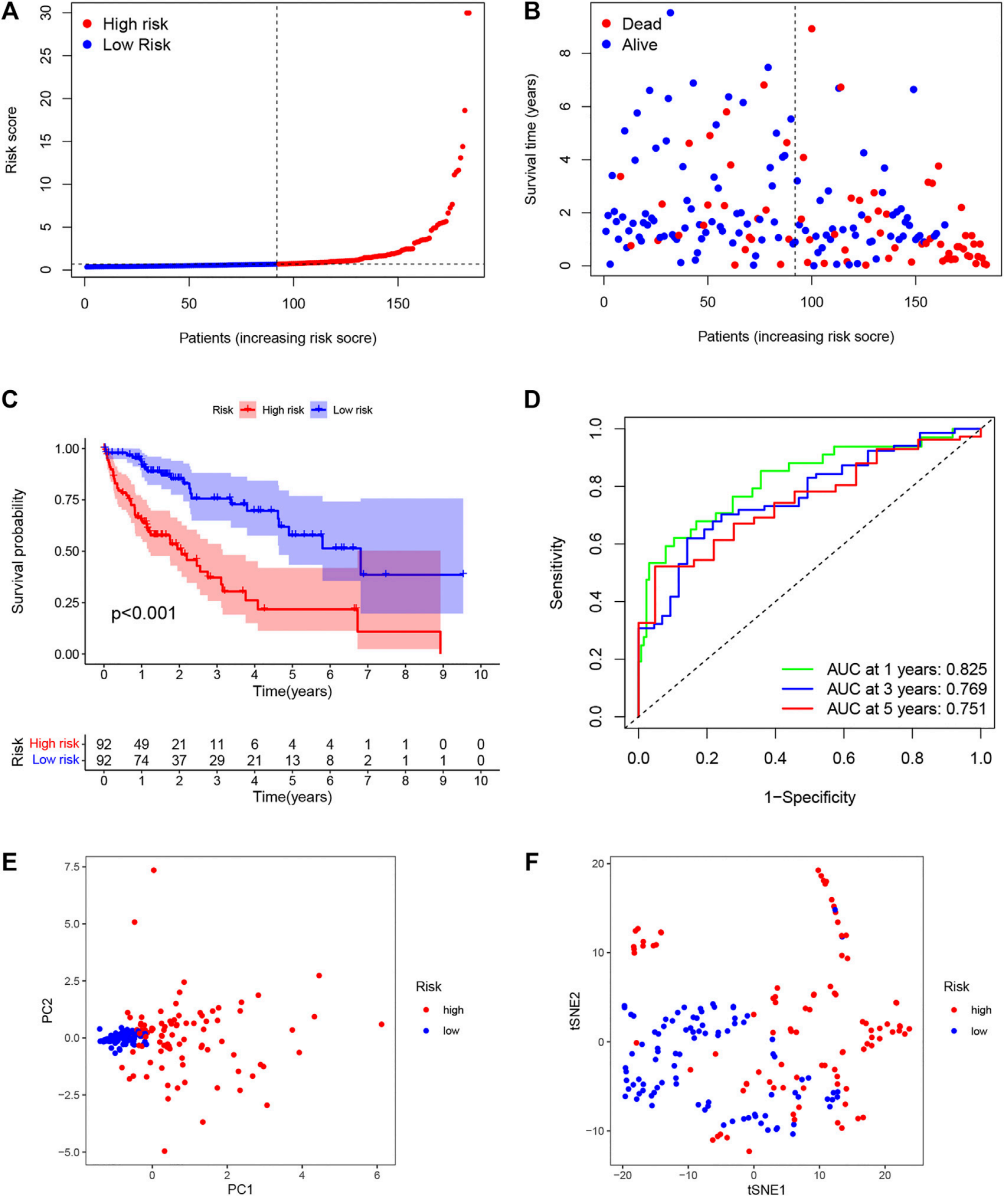
Establishment of the DENRG-based OS signature. **(A)** Distribution of training set risk scores. **(B)** OS status in the LIHC training set. **(C)** K-M analysis of OS in the two groups. **(D)** AUCs of ROC curves in training set. **(E)** PCA plot showing the significant difference between the two groups in the training set. **(F)** t-SNE uses dimensionality reduction to show the difference between the two groups in the training set.

The univariate analysis showed that 142 DENRGs were associated with OS (Figure 3A). In the subsequent LASSO analysis aimed at reducing the risk of overfitting (Figures 3B, C), 19 DENRGs were found to be associated with OS. The multivariate Cox regression narrowed this count to eight DENRGs, which were then used to construct the OS prognostic signature. The coefficients of these eight DENRGs were verified at the same time and were used to evaluate the scores for individual patients; these scores were then used to divide the patients in the training set into two groups according to risk. The scores were illustrated by risk curves and scatterplots and these, together with the patient survival data, are shown in Figure 4 A and B. K-M analysis found significantly reduced OS in high-risk individuals (Figure 4C). To assess the performance of the signature, ROC curves for 1-year, 3-year, and 5-year OS were created, and the AUCs at these time points were 0.825, 0.769, and 0.751, respectively (Figure 4D). Moreover, PCA and t-SNE analyses showed separation between the patients in the two groups (Figures 4E, F).

### Validation of the DENRG-Based Signature

**FIGURE 5:**
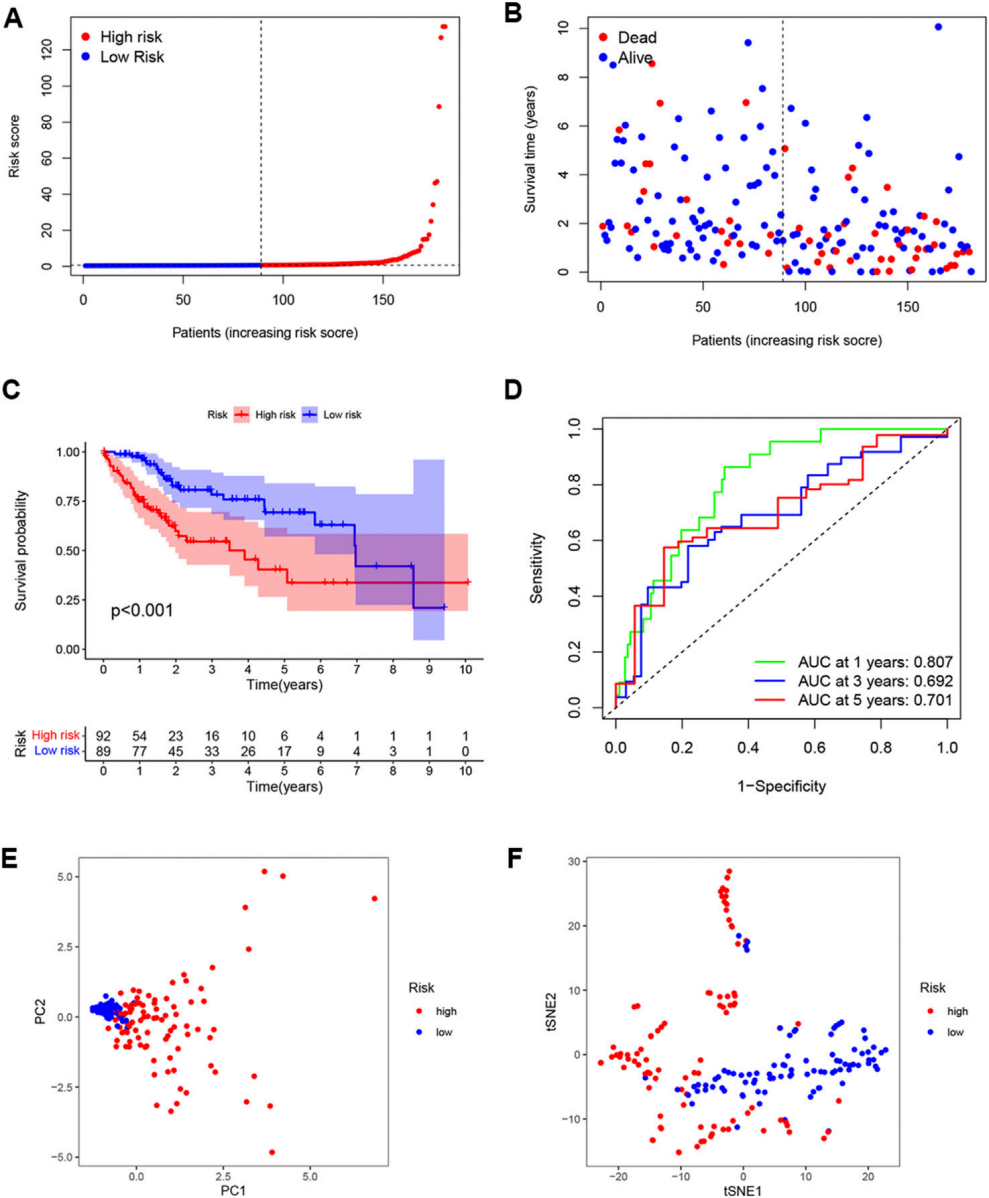
External validation of OS signature. **(A)** Distribution of validation set risk scores. **(B)** OS status in the LIHC validation set. **(C)** K-M analysis of OS in the two groups. **(D)** AUCs of ROC curves in the validation set. **(E)** PCA plot showing the significant difference between the two groups in the validation set. **(F)** t-SNE uses dimensionality reduction to show the difference between the two groups in the validation set.

Individuals in the validation set were likewise assigned to two groups according to the risk score. Of the 181 individuals in the set, 92 were classified as having high risk, and 89 were classified as having low risk. As was observed in the training set, the risk curves and scatterplots showed an association between high-risk scores and reduced OS (Figures 5A, B), while low risk was related to higher OS (Figure 5C). The AUCs for the 1-year, 3-year, and 5-year time points were 0.807, 0.692, and 0.701, respectively (Figure 5D). Furthermore, PCA and t-SNE also showed significant separation in the two-dimensional plane between the groups (Figures 5E, F). These findings imply that the signature was effective for predicting LIHC prognosis.

### Correlation Between Clinicopathological Variables and the DENRG-Based Signature

**FIGURE 6:**
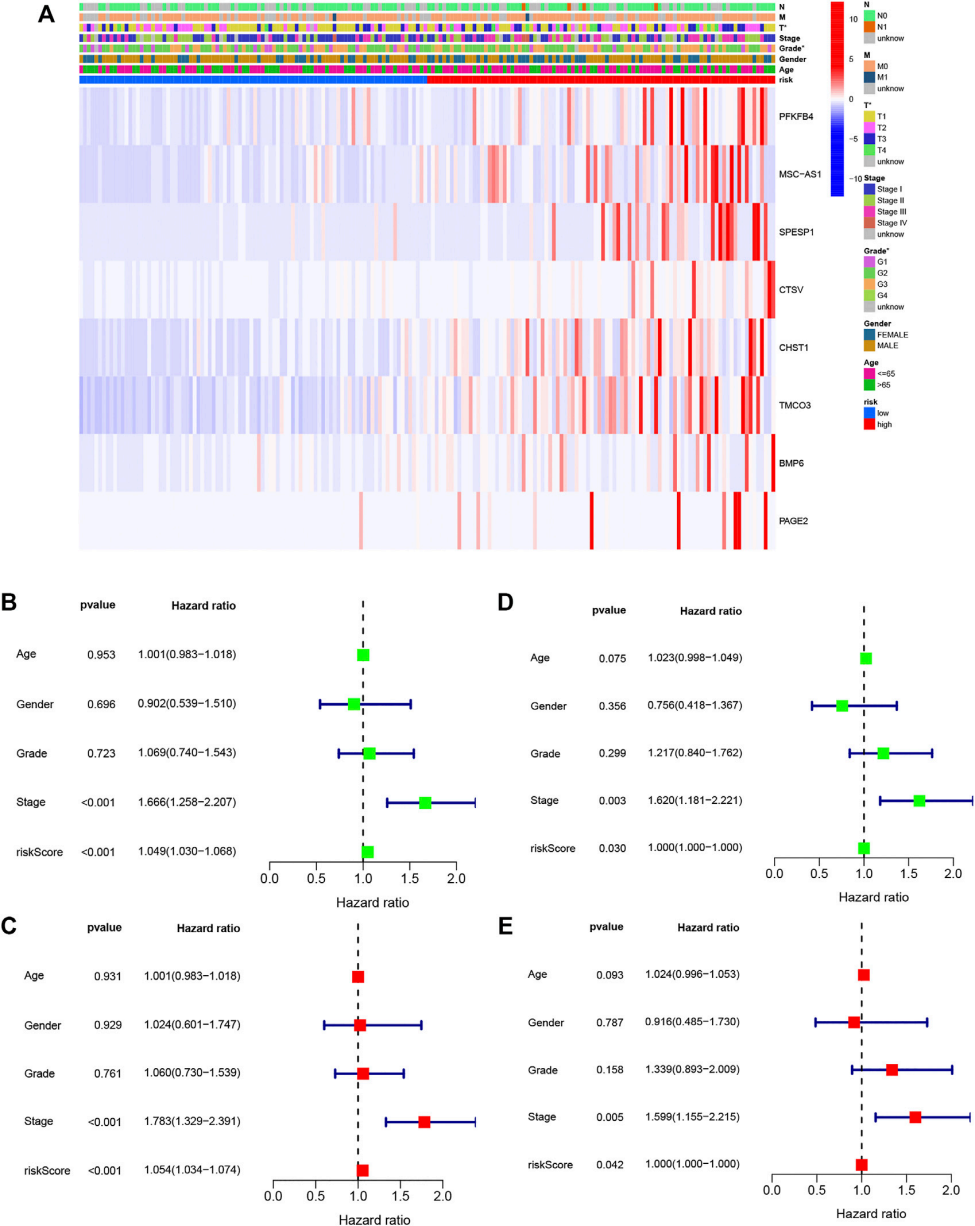
Relationships between signature risk scores and clinicopathological parameters. **(A)** Heatmap showing the expression of eight DENRGs in terms of the OS signature and clinicopathological characteristics. **(B)** Univariate analysis of all parameters of the training set OS. **(C)** Multivariate analysis of all parameters of the training set OS. **(D)** Univariate analysis of all parameters of the validation set OS. **(E)** Multivariate analysis of all parameters of the validation set OS. *p < 0.05.

The relationships between the DENRG-based signature and clinicopathological variables were examined by correlation analysis. As seen in the heatmap (Figure 6A), tumor grade and T stage were found to be significantly associated with the risk score. Compared with the low-risk group, all eight DENRGs were highly expressed in the high-risk group and were risk factors for liver cancer. Typical immunohistochemical images of five genes were downloaded from Human Protein Atlas; however, no information was available for PFKFB4, MSC-AS1, and CHST1, which were unavailable from the database (Supplementary Figure S3). Moreover, we analyzed the expression of the eight DENRGs in several other common tumors and found that except for SPESP1, these genes were highly expressed in tumors (Supplementary Figure S4). Highly expressed genes are often risk factors for tumors, which is consistent with our results. Using both univariate and multivariate Cox regression, we observed a significant link between tumor stage and the signature in the training set (Figures 6B, C) and validation set (Figures 6D, E), indicating that these can be used as independent prognostic factors for survival, and further verifying the reliability and effectiveness of the eight-gene signature we established.

### Nomogram Construction and Validation

**FIGURE 7:**
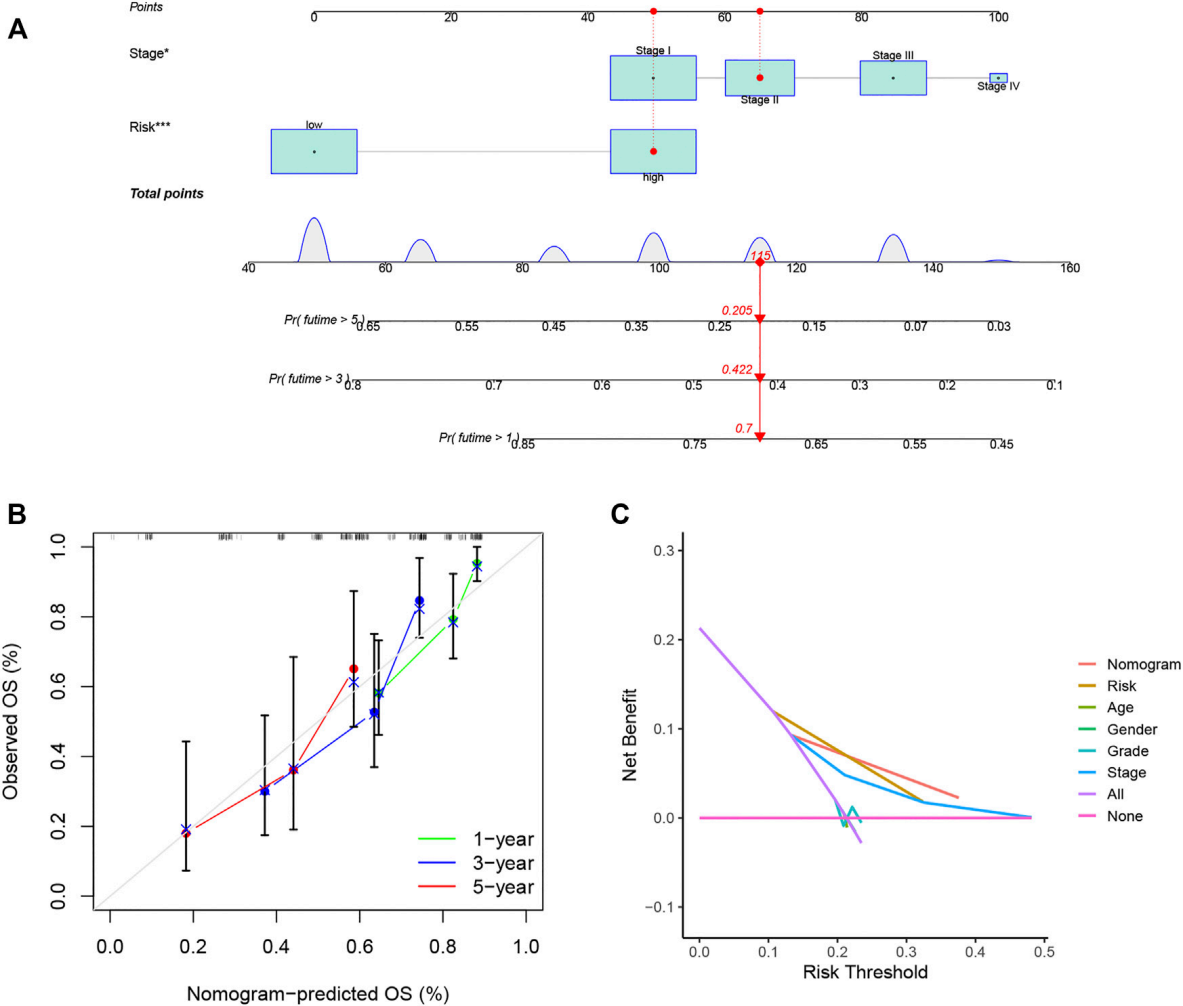
Nomograms for LIHC OS prediction. **(A)** Nomograms combining the OS signature and three clinical variables of LIHC patients in the training set. **(B)** Calibration curve for assessment of the reliability of OS prediction by the nomogram. **(C)** DCA curve showing the clinical utility of the signature. *p < 0.05, ***p < 0.001.

A nomogram was constructed for predicting OS at different time points in the training set (Figure 7A). It was found that the predicted OS matched the actual OS, shown by the calibration chart (Figure 7B). Furthermore, a DCA curve indicated that the nomogram was highly reliable in terms of individual parameters (Figure 7C). More meaningfully, the nomogram permitted the estimation of the OS of specific patients by entering the score of individual parameters, which greatly improves its practical application.

### DENRG Enrichment Analysis

GO and KEGG analyses were used to examine the underlying mechanisms for the differences seen in prognosis between the groups. The GO results indicated that these genes were associated with “extracellular matrix organization,” “phagocytosis,” and “leukocyte chemotaxis”, while KEGG demonstrated enrichment in several pathways associated with carcinogenesis, including “PI3K-AKT signaling pathway,” “HIF-1 signaling pathway,” and “TNF signaling pathway”, as well as immune-related pathways, such as “IL-17 signaling pathway” and “intestinal immune network for IgA production”. These results suggested that DENRGs are involved not only in the tumor but also in the tumor microenvironment.

### Relationships Between the DENRG Signature and the Tumor Microenvironment

**FIGURE 8:**
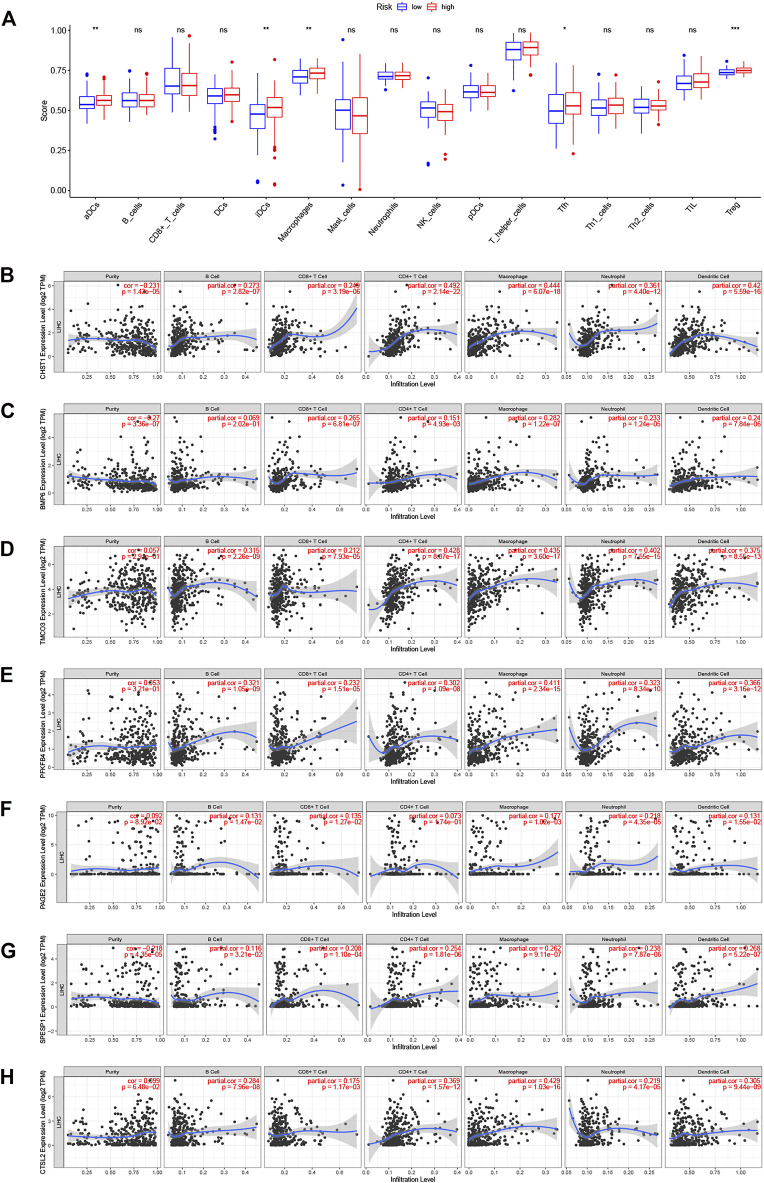
Relationships between seven DERNGs and immune infiltration. **(A)** Boxplot comparison of immune cell infiltration in the two groups. **(B–H)** Partial correlation analysis of DENRGs expression levels, tumor purity, and infiltration degree by TIMER database. **(B)** CHST1 **(C)** BMP6 **(D)** TMCO3 **(E)** PFKFB4 **(F)** PAGE2 **(G)** SPESP1 **(H)** CTSV(CTSL2). *p < 0.05, **p < 0.01, and ***p < 0.001.

Variations in immune infiltration can result in varied clinical outcomes despite the presence of the same histological cancer type (Li et al., 2016). As the KEGG analysis showed enrichment in immune pathways, we investigated possible variations in immune infiltration in the training-set groups. This found higher infiltration levels in the high-risk patients, specifically, infiltration of dendritic cells, macrophages, T follicular helper cells, and regulatory T cells (*p* < 0.05) (Figure 8A). TIMER was then used to examine the associations between seven DENRGs (TIMER does not contain information on non-coding RNAs; thus, MSC-AS1 was excluded), tumor purity, and immune infiltration. This indicated associations between all seven DENRGs and the levels of six cell types (Figures 8B–H). In addition, we analyzed the expression of immune checkpoints (Supplementary Figure S5A) and m6a regulators (Supplementary Figure S5B) and found that the expression of 33 immune checkpoints, including CD274 (PD-L1), and most m6a regulators differed significantly between the high- and low-risk groups. These findings indicate that these DENRGs may be closely linked to the formation of the tumor microenvironment and thus may represent immunotherapeutic targets for LIHC.

### Survival Analysis Based on the Expression of Eight Genes

**FIGURE 9:**
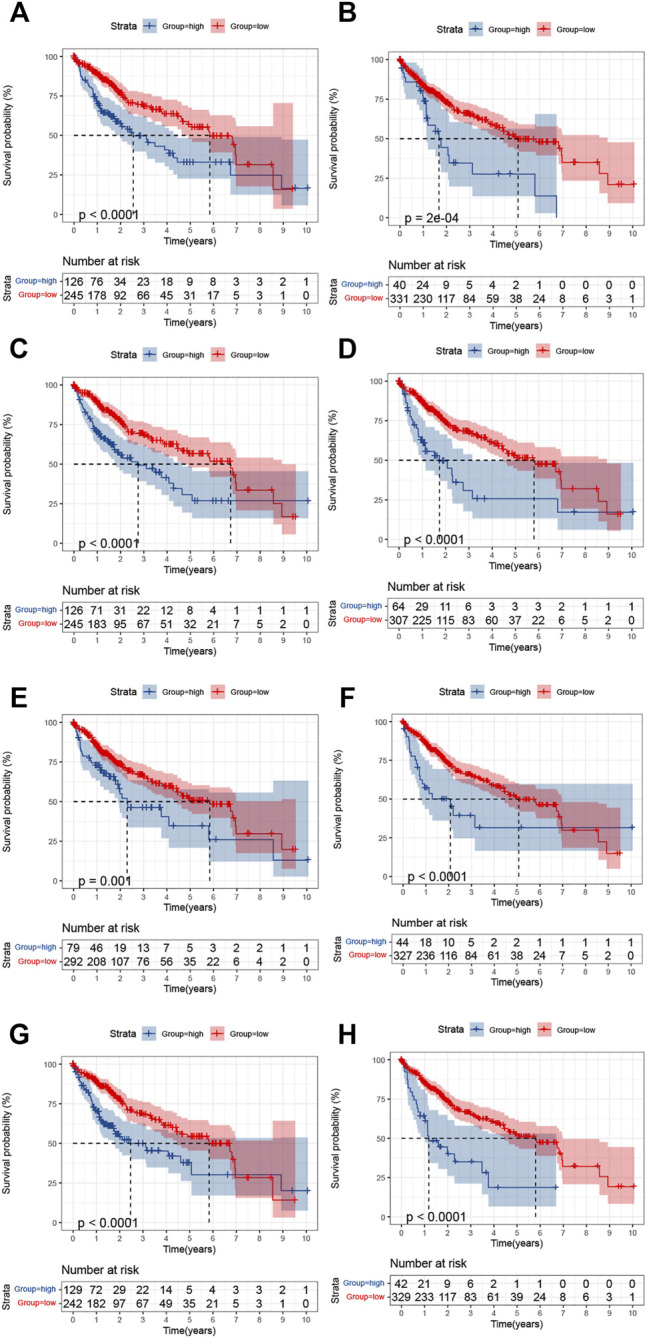
Kaplan-Meier analysis of the survival in the high- and low-expression groups of the eight DENRGs. **(A)** CHST1 **(B)** BMP6 **(C)** TMCO3 **(D)** PFKFB4 **(E)** PAGE2 **(F)** SPESP1 **(G)** CTSV(CTSL2) **(H)** MCS-AS1.

Data on the expression of PFKFB4, MSC-AS1, SPESP1, BMP6, CHST1, CTSV (CTSL2), TMCO3, and PAGE2 were obtained for LIHC patients. The optimal cutoff value in terms of survival prediction was confirmed by using X-tile software. K-M survival analysis shows that the survival prognoses in the high-expression groups for the genes were relatively worse than those for low-expression groups (Figures 9A–H). This is consistent with the identification of these genes as high-risk factors (Figure 6A), verifying the accuracy of the signature.

## Discussion

Intratumor heterogeneity brings new challenges to tumor treatment and is a key factor in the failure of cancer treatment (Meacham and Morrison, 2013; Janku, 2014). LIHC, the most widespread primary liver malignancy, is a heterogeneous tumor, with significant variation in both the immune microenvironments and angiogenic characteristics of tumors (Jeng et al., 2015). The efficacy of immunotherapy is highly variable, with some patients responding well and others only to a limited extent (Zhou et al., 2009). However, at present, the molecular mechanisms underlying these clinical differences in patients are unclear. Our research on necroptosis may provide a possible explanation for exploring the different results of tumor immunotherapy.

Necroptosis, originally discovered as a substitute for apoptosis involving the participation of death domain receptors (Degterev et al., 2005), is a process of cellular self-destruction activated by extracellular signals (death receptor-ligand binding) or intracellular signals (the presence of foreign microbial nucleic acids) when apoptosis is blocked (Galluzzi et al., 2018; Frank and Vince, 2019). Necroptosis differs from necrosis and other forms of programmed cell death such as apoptosis. Apoptosis represents an active self-destructive death of cells that is controlled by specific genes and is not affected by external stimuli (Galluzzi et al., 2018). Necrotic cell death occurs when pathological stimuli (hypoxia, physical, chemical, biological factors, or the immune response) lead to irreversible cellular damage and is essentially a passive process (Gogna et al., 2012). HMGB1 is released by necrotic cells and is considered a marker of necrosis (Scaffidi et al., 2002). HMGB1 has been reported to play an important role in multiple pathological processes of liver cancer, including proliferation (He et al., 2010), migration (Xiao et al., 2014), cell differentiation (Kostova et al., 2010), and the inflammatory response (Huebener et al., 2015). This suggests that there is a close link between necrosis and the development of LIHC. However, there is little in-depth research on the relationship between necroptosis and LIHC. Necroptosis does not depend on caspase activity but requires phosphorylation of MLKL by RIPK3 (Sun et al., 2012; Zhao et al., 2012). This phosphorylation event leads to the oligomerization and membrane association of MLKL, resulting in the secretion of DAMPs, cellular swelling, and membrane rupture (Frank and Vince, 2019). A recent study by Snyder et al. found that injecting necroptotic cells into mouse tumors guided killer T cells to attack the malignant tumor and slow its growth. In addition, an enzyme that induced necroptosis, initiating tumor shrinkage, was described (Snyder et al., 2019). It has also been reported that nanobubbles resulting from necroptosis affected tumor immunity by stimulating dendritic cell maturation and cytotoxic T cell activation (Um et al., 2020). Similarly, vaccination with necroptotic cancer cells was found to stimulate the cross-priming of cytotoxic CD8a+ T cells and to produce IFN-g *ex vivo*, generating a strong anti-tumor response (Aaes et al., 2016). These strategies may improve the efficacy of existing immunotherapy, and also indicate that there is a tight connection between necroptosis and immunity in tumors.

We then explored necroptosis in relation to both tumorigenesis and the immune microenvironment and used necroptosis-related genes to create a risk signature for prognostic prediction in LIHC patients. We first performed an unsupervised cluster classification of LIHC patients centered on the levels of known necroptotic regulators. This showed that not only clinical variables such as T staging and sex but also immune scores and matrix scores had significantly different distributions between the C1 and C2 clusters (Figure 2C). As unsupervised learning algorithms can identify natural groupings based on the inherent features of the data (Lenning et al., 2017; Bobić et al., 2019), we can speculate that there is an intrinsic relationship between necroptosis and the immune microenvironment in LIHC.

Next, 1,516 DENRGs were identified between the C1 and C2 patients, representing candidate genes for the signature construction. We analyzed these DENRGs using univariate, LASSO, and multivariate analyses to identify the most suitable genes for the signature. Both survival distribution and K-M curves indicated significant differences in OS between the high and low risk patients. ROC curves showed the satisfactory predictive capability of the signature. Furthermore, the signature was successfully validated with an equally satisfactory result in the validation set.

Eight genes (PFKFB4, MSC-AS1, SPESP1, CTSV, CHST1, TMCO3, BMP6, and PAGE2) were incorporated into the final prognostic signature. It is reported that 6-phosphofructo-2-kinase/fructose-2,6-bisphosphatase 4 (PFKFB4) is a metabolic enzyme that mediates the proliferation, progression, and drug resistance of a variety of tumors through glucose metabolism (Dasgupta et al., 2018; Gao et al., 2018; Feng et al., 2021; Shen et al., 2021). Musculin antisense RNA 1 (MSC-AS1) is a lncRNA, which interacts largely with miRNAs and acts as an oncogene in several cancers (Li et al., 2021; Liu et al., 2021; Ma et al., 2021; Zhao et al., 2021). Cathepsin V (CTSV/CTSL2) is a proteolytic enzyme that degrades the extracellular matrix promoting tumor metastasis and invasion (Lin et al., 2020; Toss et al., 2020; Wang et al., 2020). Bone morphogenetic protein 6 (BMP6) is involved in growth, differentiation, and apoptosis and is an important regulator of tumors and the tumor microenvironment (Liu et al., 2014; Seo et al., 2019; Stieglitz et al., 2019; García Muro et al., 2021). However, there are few reports on the roles of SPESP1, CHST1, TMCO3, and PAGE2 in tumors. At present, none of these genes has been associated with necroptosis, which will be an important direction for our future research.

To enable the clinical use of the signature, we created a nomogram to predict the 1-year, 3-year, and 5-year OS. Univariate and multivariate analyses demonstrated that the risk score was an independent prognostic factor for prognosis prediction in both the training and validation sets. Comparison between the prediction efficacy of the nomogram and clinical parameters demonstrated the effectiveness of the nomogram for outcome prediction. The calibration curve confirmed a high degree of fit. All these results verified the accuracy of the nomogram.

**FIGURE 10:**
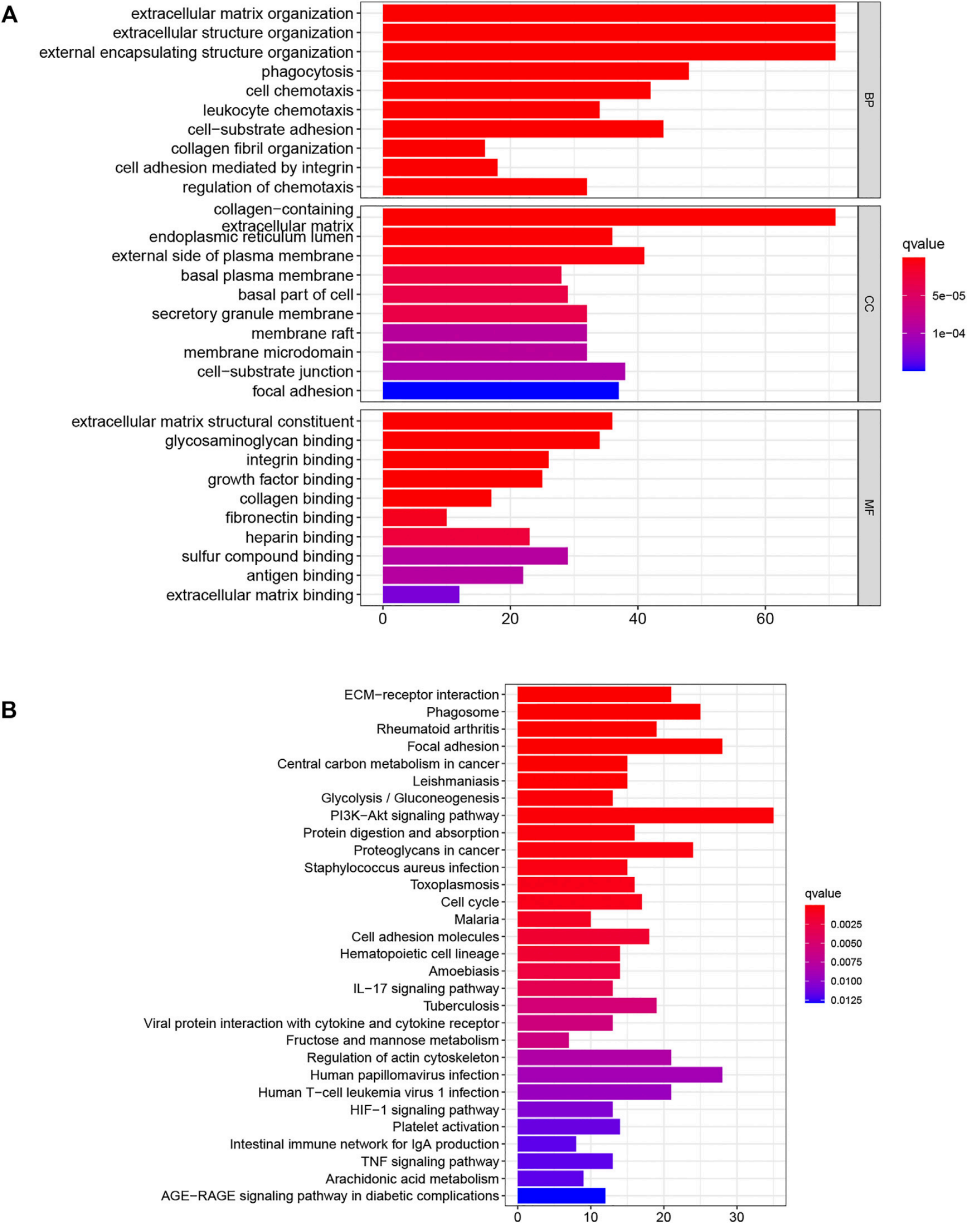
DENRG enrichment in the training set **(A)** GO **(B)** KEGG. BP Biological process, CC Cellular component, MF Molecular function.

Investigation into DENRG enrichment between the groups was used to examine the underlying mechanisms responsible for the poor outcomes in the high-risk patients. As can be seen from Figure 10, enrichment of several classic cancer-associated pathways (including the PI3K-AKT and TNF signaling pathways) and immune-associated pathways (for example, IL-17 signaling pathway) was found suggesting that prognosis may be linked to the immune microenvironment. We thus investigated the links between the signature and immune cells. Our results support the observations of McDermott et al. (2018) and Thompson et al. (2007), showing associations between high immune cell infiltration and poor prognosis. The finding by Lu et al. (2021) that p75NTR/proBDNF modulates the immune microenvironment via necroptosis further confirms the close connection between necroptosis and immunity. This implicates both necroptosis and the tumor microenvironment in the prediction of LIHC prognosis.

## Conclusion

In our study, we developed and validated an accurate signature based on eight DENRGs. Integration of the signature with clinical parameters was effective in predicting the prediction of LIHC patients. The signature showed that necroptosis was associated with the tumor microenvironment, and that immune cell infiltration was linked to poor prognosis. Our findings provide a possible explanation for the different prognoses of patients and provide new directions for identifying markers and treatment targets.

## Data Availability

The original contributions presented in the study are included in the article/Supplementary Material, further inquiries can be directed to the corresponding author.
