# Supplementary figures and images for: Construction and Validation of a Necroptosis-Related Signature Associated With the Immune Microenvironment in Liver Hepatocellular Carcinoma
Source: Front Genet. 2022 Apr 11;13:859544. doi: 10.3389/fgene.2022.859544 (PMC9037783; doi:10.3389/fgene.2022.859544)

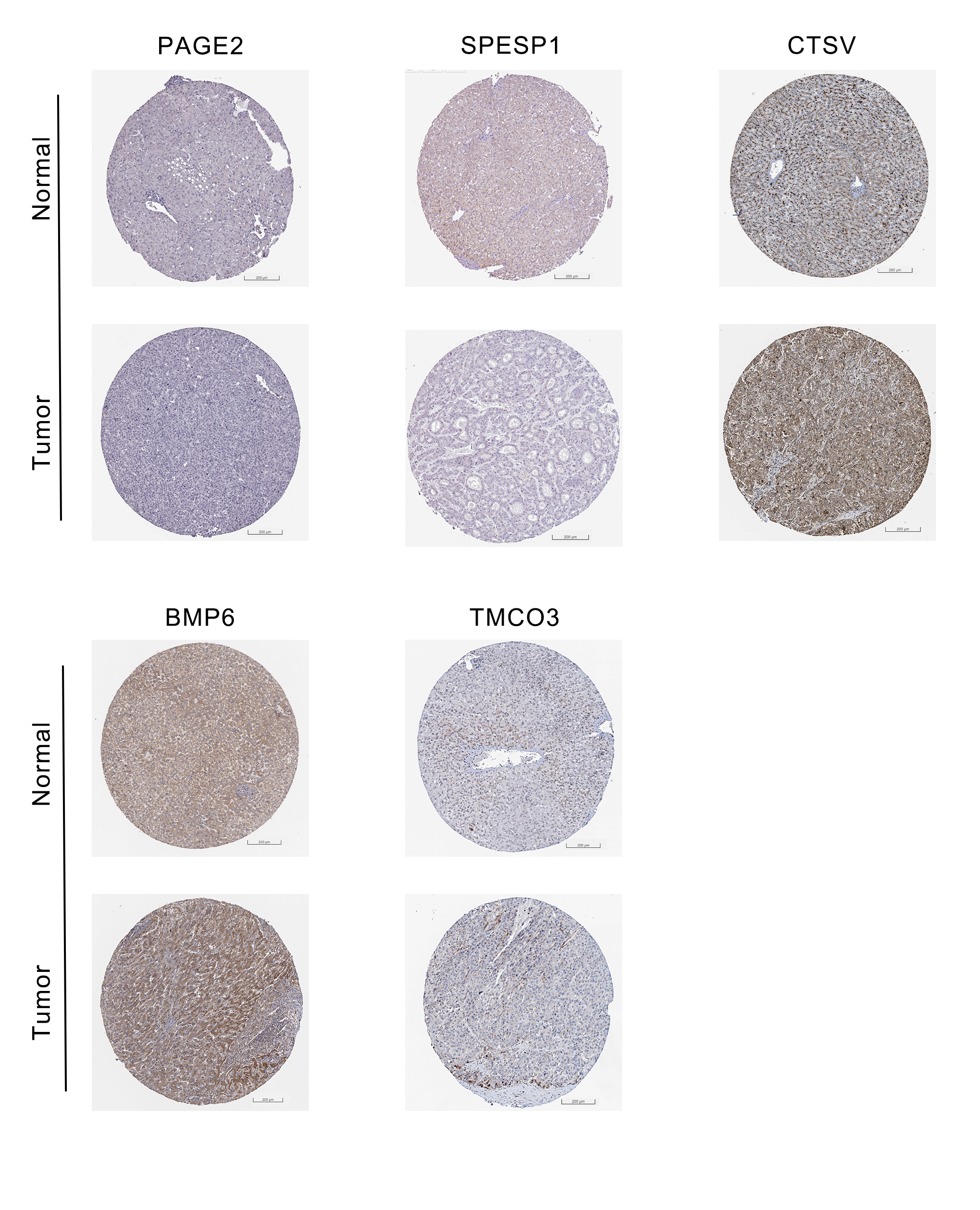

Supplement: Supplementary file 2 [file Image3.TIF]

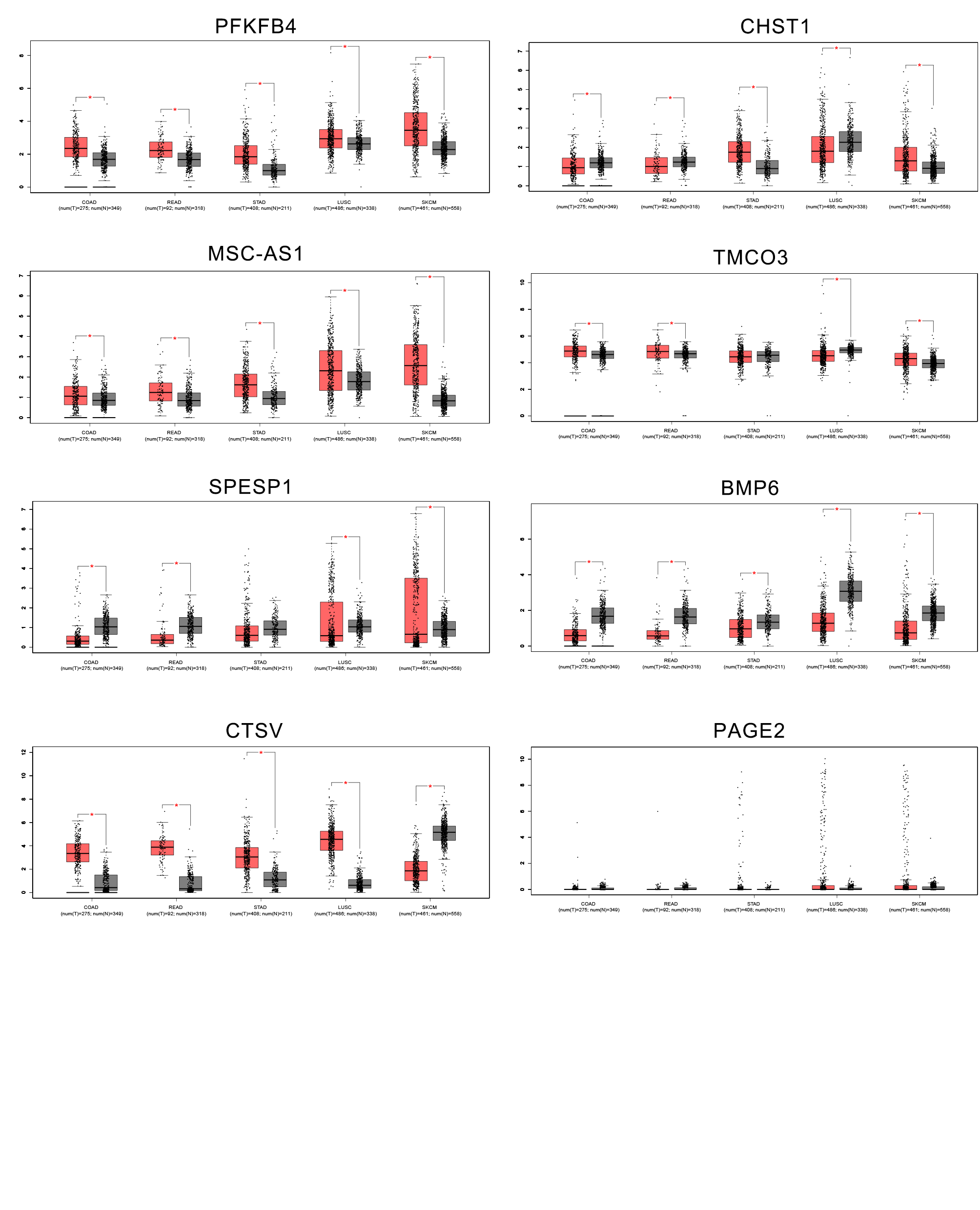

Supplement: Supplementary file 3 [file Image4.TIF]

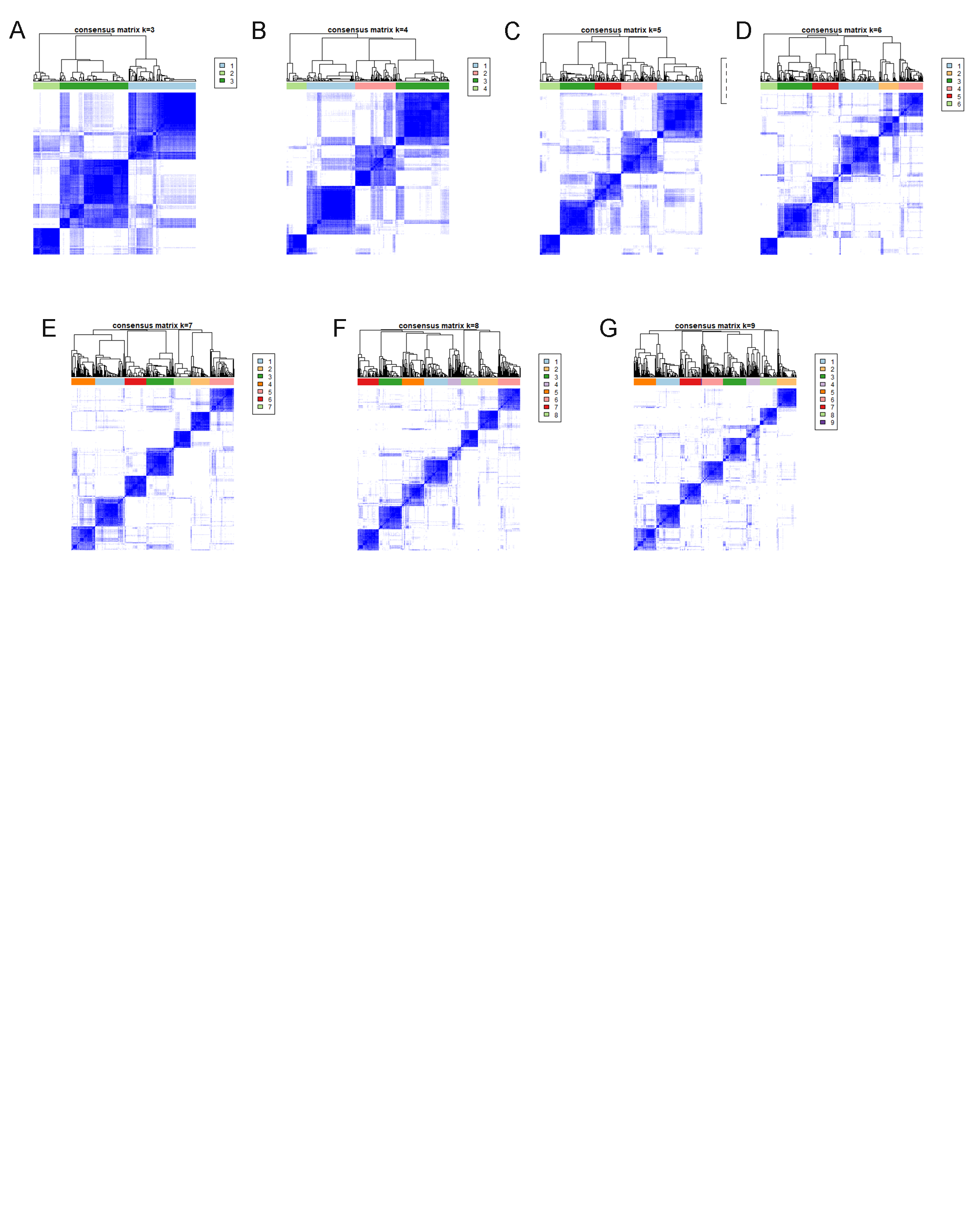

Supplement: Supplementary file 4 [file Image2.TIF]

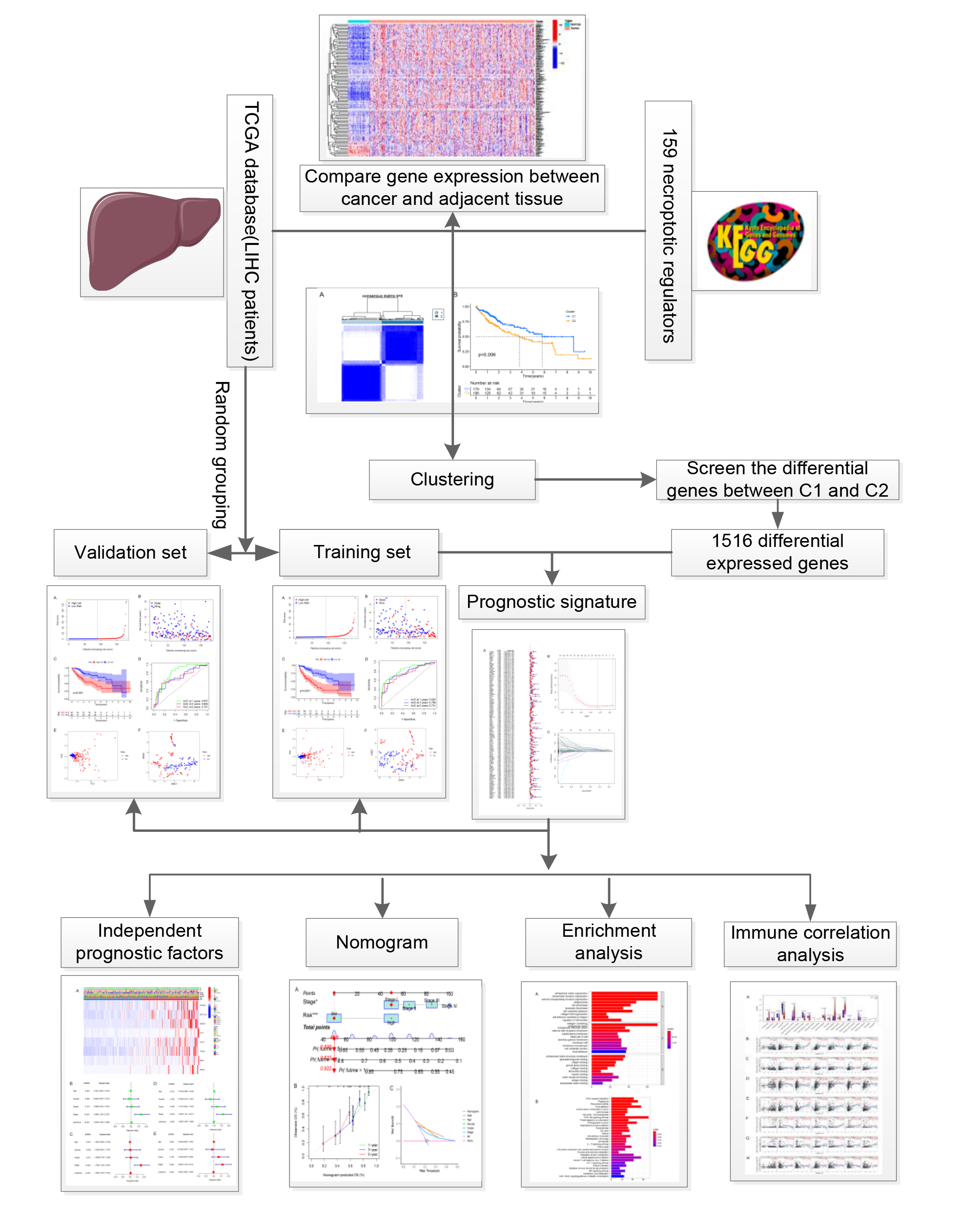

Supplement: Supplementary file 5 [file Image1.TIF]

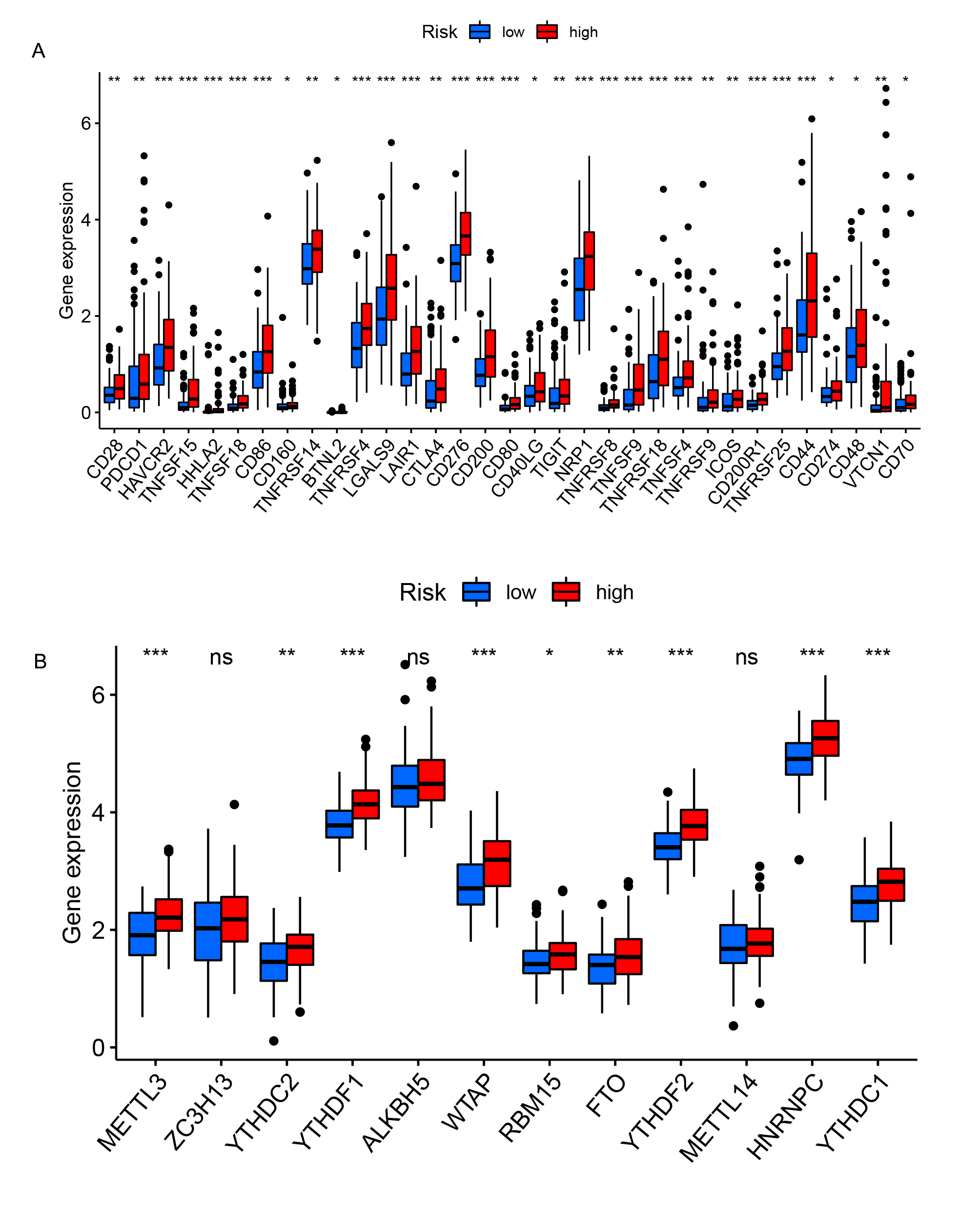

Supplement: Supplementary file 7 [file Image5.TIF]
